# Association of Resistin Gene Polymorphisms with Oral Squamous Cell Carcinoma Progression and Development

**DOI:** 10.1155/2018/9531315

**Published:** 2018-10-14

**Authors:** Wei-Hung Yang, Shoou-Jyi Wang, Yung-Sen Chang, Chen-Ming Su, Shun-Fa Yang, Chih-Hsin Tang

**Affiliations:** ^1^School of Chinese Medicine, China Medical University, Taichung, Taiwan; ^2^Department of Nursing, National Taichung University of Science and Technology, Taichung, Taiwan; ^3^Department of Orthopedic Surgery, Taichung Hospital, Ministry of Health and Welfare, Taichung, Taiwan; ^4^Department of Orthopedic Surgery, Chang-Hua Hospital, Ministry of Health and Welfare, Puhsin Township, Changhua County, Taiwan; ^5^Department of Biomedical Sciences Laboratory, Affiliated Dongyang Hospital of Wenzhou Medical University, Dongyang, Zhejiang, China; ^6^Institute of Medicine, Chung Shan Medical University, Taichung, Taiwan; ^7^Department of Medical Research, Chung Shan Medical University Hospital, Taichung, Taiwan; ^8^Department of Pharmacology, School of Medicine, China Medical University, Taichung, Taiwan; ^9^Graduate Institute of Basic Medical Science, China Medical University, Taichung, Taiwan; ^10^Chinese Medicine Research Center, China Medical University, Taichung, Taiwan; ^11^Department of Biotechnology, College of Health Science, Asia University, Taichung, Taiwan

## Abstract

Oral squamous cell carcinoma (OSCC) accounts for over 90% of malignant neoplasms of the mouth. In Taiwan, OSCC is the fourth most common male cancer and the fourth leading cause of male cancer death. Resistin (*RETN*) is an adipokine that is associated with obesity, inflammation, and various cancers. Here, we examine the association between four single nucleotide polymorphisms (SNPs) of the* RETN* gene (rs3745367, rs7408174, rs1862513, and rs3219175) and OSCC susceptibility as well as clinical outcomes in 935 patients with OSCC and in 1200 cancer-free healthy controls. We found that, in 1465 smokers,* RETN* polymorphisms carriers with the betel-nut chewing habit had a 6.708–10.882-fold greater risk of having OSCC compared to* RETN* wild-type carriers without the betel-nut chewing habit. Patients with OSCC who had A/A homozygous of* RETN* rs3219175 polymorphism showed a high risk for an advanced tumor size (> T2), compared to those patients with G/G homozygotes. In addition, A/T/G/G haplotype significantly increased the risks for OSCC by 1.376-fold. This study is the first to examine the risk factors associated with* RETN* SNPs in OSCC progression and development in Taiwan.

## 1. Introduction

Oral squamous cell carcinoma (OSCC) accounts for over 90% of malignant neoplasms of the mouth [1]. In Taiwan, OSCC is the fourth most common male cancer and the fourth leading cause of male cancer death [2]. Despite combination treatment with radiation, surgery, and chemotherapy, the prognosis of OSCC remains poor [3, 4]. OSCC occurs through multiple genetic alterations due to chronic exposure to environmental carcinogens. Chronic inflammation, alcohol and tobacco consumption, betel quid chewing, and viral infection are all implicated as risk factors for OSCC [5–7]. Single nucleotide polymorphisms (SNPs) constitute the most common type of DNA sequence variation influenced gene expression and protein production and function as well as disease susceptibility in particular individuals [8, 9]. Previous research has suggested that gene polymorphisms may increase the risk of developing oral cancer. For instance, individuals carrying specific C-C chemokine ligand 4 (*CCL4*), interleukin-18 (*IL-18*), or intercellular adhesion molecule-1 (*ICAM-1*) SNPs have a higher susceptibility to OSCC compared with wild-type controls [2, 10, 11]. Thus, identifying genes that impact upon OSCC susceptibility is important for early detection of disease.

Resistin is a 12.5-kDa cysteine-rich adipokine that is constitutively secreted by adipose tissue [12]; resistin levels in plasma correlate with inflammatory markers and coronary artery calcification, a measure of coronary atherosclerosis [13]. Several SNPs have been discovered in the* RETN* promoter and 3′-untranslated regions [14]. Genetic variation in* RETN* is associated with a greater risk of various diseases, including metabolic syndrome and colon cancer [15, 16], while a functional* RETN* gene polymorphism at -420 (rs186513) appears to promote susceptibility to type 2 diabetes [17] and* RETN* SNPs have been found to correlate with lung cancer progression in a Chinese Han population [18]. Moreover, upregulation of resistin gene expression in human breast cancer tissues has been reported [19]. Up until now, no correlation has been established between* RETN* gene polymorphisms and OSCC prognosis. This case-control study sought to determine the role of four* RETN* SNPs and clinicopathological features in OSCC susceptibility in a cohort of Taiwan individuals.

## 2. Materials and Methods

### 2.1. Participants

We enrolled 935 patients (cases) presenting with OSCC to Chung Shan Medical University Hospital in Taichung or Changhua Christian Hospital in Changhua, Taiwan, between 2007 and 2015. A total of 1,200 anonymized healthy controls were randomly selected from the Taiwan Biobank Project; none had a previous history of cancer at any sites. The study exclusion criteria specified that subjects with oral precancerous disease, such as oral submucous fibrosis, leukoplakia, erythroplakia, or verrucous hyperplasia, were ineligible for enrollment. Data pertaining to primary tumor size, lymph node involvement, and histologic grade were extracted from medical records. Tumors were staged according to the American Joint Committee on Cancer (AJCC) Tumor, Node, Metastasis (TNM) staging criteria [20]. Prior to enrollment, all participants provided informed written consent and completed a structured questionnaire surveying sociodemographic data and cigarette and alcohol use.

### 2.2. Determination of Genotypes

Total genomic DNA was isolated from whole blood specimens using QIAamp DNA Blood Mini Kits (Qiagen, Valencia, CA), as per the manufacturer's instructions. DNA was dissolved in TE buffer (10 mM Tris pH 7.8, 1 mM EDTA) and stored at −20°C until quantitative polymerase chain reaction (PCR) analysis. Four* RETN* SNPs were selected (rs3745367, rs7408174, rs1862513, and rs3219175) with minor allele frequencies >5% in the HapMap population. Primers and probes consisted of rs3745367 “CTCCGACTGTCCCCACCTTATCCAC[A/G]GCTCCA­AACCCAA”, rs7408174 “TTTTACCACAAAAAGGCC­CGTTGTA[C/T]TGGAAACAAAGAA”, rs1862513 “CCT­GACCAGTCTCTGGACATGAAGA[C/G]GGAGGCCCT­GTTG”, rs3219175 “CTCCAGCCCTTACTGTCTGCTCAG­G[A/G]GCTTCCTCTTGGC”. These SNPs have previously been found to correlate with progression of lung and breast cancer as well as development of rheumatoid arthritis [18, 19, 21]. We genotyped the SNPs with the commercially available TaqMan SNP genotyping assay (Applied Biosystems, Warrington, UK), according to the manufacturer's protocols [22, 23].

### 2.3. Statistical Analysis

The genotype distribution of each SNP was analyzed for Hardy-Weinberg equilibrium and confirmed by Chi-square analysis. Demographic characteristics were compared between patients and controls using the Mann–Whitney U-test and Fisher's exact test. Associations between genotypes, OSCC risk, and clinicopathological characteristics were estimated using adjusted odds ratios (AORs) and 95% confidence intervals (CIs) obtained from age- and gender-adjusted multiple logistic regression models. The statistical analysis about haplotype was according to previous studies [24, 25]. A *p* value of <0.05 was considered statistically significant. Data were analyzed using SAS statistical software (Version 9.1, 2005; SAS Institute Inc., Cary, NC).

## 3. Results

Mean age did not differ significantly between men with (n=935) and without OSCC (n=1,200) (Table 1). Significant between-group differences were found for betel quid chewing (*p*<0.001), cigarette smoking (*p*<0.001), and alcohol drinking (*p*<0.001); all behaviors were significantly more prevalent among the OSCC cohort compared with controls (Table 1). Around half of the patients (49.8%) had stage I/II disease and half (50.2%) had stage III/IV disease (Table 1). One-third (32.8%) had N1–N3 lymph node metastasis. Nearly all tumors (99.0%) were classified as M0 status; the majority of tumors (85.1%) were moderately and poorly differentiated (Table 1).

Table 2 shows the* RETN* genotypes in OSCC patients and controls. In the controls, all genotypic frequencies were in Hardy-Weinberg equilibrium (*p*>0.05). In both patients and controls, most of those with the rs3745367 SNP were homozygous for the G/G genotype, most of those with the rs7408174 SNP were homozygous for the T/T genotype, most of those with the rs1862513 SNP were homozygous for G/G, and most of those with the rs3219175 SNP were homozygous for G/G (Table 2). After adjusting for potential confounders, we found no significant differences in the incidences of OSCC patients with the rs3745367, rs7408174, rs1862513, and rs3219175 polymorphisms compared to controls.

Tobacco consumption and betel quid chewing are important risk factors for the development of OSCC [5–7]. The effect of the interaction between tobacco consumption and betel quid chewing with* RETN* genotypes on OSCC progression is shown in Table 3. Among all 1,465 smokers (healthy controls and patients combined), those who either had at least one A allele of rs3745367, one C allele of rs74081741, one C allele of rs1862513, one A allele of rs3219175 or chewed betel nuts were 5.838-fold (95% CI: 3.985-8.552), 4.105-fold (95% CI: 2.960-5.692), 5.090-fold (95% CI: 3.493-7.418), and 4.134-fold (95% CI: 3.028-5.644) more likely, respectively, to develop OSCC compared to smokers with wide-type homozygotes who did not chew betel nuts. Moreover, smokers with at least one A allele of rs3745367, one C allele of rs74081741, one C allele of rs1862513, or one A allele of rs3219175 and who chewed betel nuts had respective risks that were 8.544-fold (95% CI: 5.668-12.880), 8.583-fold (95% CI: 6.026-12.225), 6.708-fold (95% CI: 4.467-10.074), and 10.882-fold (95% CI: 7.861-15.064) higher, respectively, for developing OSCC compared with the wild-type homozygous smokers. These results suggest that* RETN* gene polymorphisms have a strong impact upon oral cancer susceptibility in men who smoke tobacco and/or chew betel nuts.

Next, we compared associations between the* RETN* rs3219175 polymorphism and clinical status in OSCC patients aged >54 years. Compared with patients with the G/G genotype, those with the A/A genotype at the rs3219175 SNP were 2.480-fold (95% CI: 1.058-5.814) more likely to develop large tumors (>T2) (Table 4). No significant between-group differences were observed for clinical stage, lymph node metastasis, distant metastasis, or cell differentiation at the rs3219175 SNP (Table 4).

An evaluation of the haplotypes sought to determine the combined effect of the four polymorphisms on OSCC susceptibility. In an analysis of distribution frequencies of* RETN* rs3745367, rs7408174, rs1862513, and rs3219175 haplotypes, the most common haplotype in healthy controls was G/T/G/G, which was therefore selected as the reference. The A/T/G/G* RETN* haplotype significantly increased the risk for developing OSCC by 1.376-fold (95% CI: 1.039-1.823,* p*<0.05) (Table 5). Furthermore, A/T/G/G* RETN* haplotype also enhanced the risk for developing OSCC in those who chewed betel nuts by 18.39-fold (95% CI: 12.333-27.421,* p*<0.05) (Table 6).

## 4. Discussion

Resistin is an adipokine that is associated with obesity, inflammation, and various cancers [26–29]. Furthermore, evidence suggests that in patients with lung cancer, high serum resistin levels may play a role in the pathogenesis of cancer cachexia [30]. Upregulation of resistin in serum has been detected in OSCC patients [31].* RETN* polymorphisms have been identified in various cancers, including colon, breast, and lung [16, 18, 19], although scant data exist on the involvement of* RETN* polymorphisms in OSCC. To the best of our knowledge, this current study is the first to examine the distribution of the rs3745367, rs7408174, rs1862513, and rs3219175 SNPs and their possible association with susceptibility to the development of OSCC. We also investigated susceptibility to OSCC when these* RETN* SNPs were combined with environmental carcinogens. Interestingly, we did not find any significant differences between the frequencies of OSCC patients and controls with the rs3745367, rs7408174, rs1862513, and rs3219175 polymorphisms. We did discover that OSCC patients aged >54 years carrying the rs3219175 G/G homozygous polymorphism had a significantly high risk of developing large-size tumors compared to those carrying the rs3219175 A/A homozygous polymorphism. However, we did not recruit the survival results of OSCC patients. Future research could evaluate the association of* RETN* polymorphisms with survival of OSCC patients. In addition, it would be advisable to collect data on a larger number of patients for analysis of the functions of* RETN* polymorphisms in OSCC.

The linkage disequilibrium is expressed across the human genome and therefore could be used as a genetic marker to locate adjacent variants that participate in the detection and treatment of disease. Haplotype analyses can provide data on disease susceptibility [32]. Our evaluation of the impact of different haplotype combinations of four* RETN* SNPs (rs3745367, rs7408174, rs1862513, and rs3219175) upon the risk of OSCC revealed that the A/T/G/G haplotype is associated with a high risk for OSCC when compared with the reference haplotype, G/T/G/G. Furthermore, compared with other SNPs, the A/T/G/G* RETN* haplotype also enhanced the risk for developing OSCC in those who chewed betel nuts. We speculate that this haplotype is in linkage disequilibrium with other functional polymorphisms that are responsible for the susceptibility to OSCC.

Exposure to environmental carcinogens might lead to an earlier onset of oral cancer development. It is known that genomic changes during hepatocarcinogenesis progressively alter the hepatocellular phenotype to produce cellular intermediates that evolve into malignancy [33]. Polymorphisms of several genes are known to be associated with the risk of OSCC [34]. Thus, genetic components may play a pivotal role in carcinogenesis. Our study demonstrates a synergistic effect between betel quid chewing and tobacco smoking with four* RETN* SNPs (rs3745367, rs7408174, rs1862513, and rs3219175) on the risk of developing OSCC. Smokers with at least one A allele of rs3745367, one C allele of rs74081741, one C allele of rs1862513, or one A allele of rs3219175 and who chewed betel nuts were more likely to develop OSCC, with respective odds of 8.544 (95% CI: 5.668-12.880), 8.583 (95% CI: 6.026-12.225), 6.708 (95% CI: 4.467-10.074), and 10.882 (95% CI: 7.861-15.064). Long-term exposure to tobacco smoke and betel-nut chewing has been found to promote chronic inflammation reactions in oral tissue, subsequently leading to random genetic alteration and the development of oral cancer [35]. We suggest that tobacco smoking and betel-nut chewing in combination with* RETN* polymorphisms can increase the risk of rapid progression to oral cancer; thus, patients with a higher* RETN* polymorphism need to be aware of their higher risk of oral cancer.

Our results demonstrate an association between* RETN* gene variants and risk of OSCC. Compared with OSCC patients carrying the G/G genotype, those with the A/A genotype at the rs3219175 SNP were prone to developing large-size tumors. In an analysis of haplotype combinations of four* RETN* SNPs (rs3745367, rs7408174, rs1862513, and rs3219175), the A/T/G/G haplotype was found to be associated with a high risk of OSCC. Thus,* RETN* could serve as a genetic prognostic marker for OSCC treatment.

## Figures and Tables

**Table 1:** Demographic and clinical characteristics of 1,200 cancer-free, healthy males (controls) and 935 Taiwanese male patients with oral squamous cell carcinoma.

Variable	Controls (N=1,200)	Patients (N=935)	*p*-value
Age (years)			
≤54	566 (47.2%)	453 (48.5%)	*p*=0.556
>54	634 (52.8%)	482 (51.5%)	
Betel quid chewing			
No	1,001 (83.4%)	190 (20.3%)	
Yes	199 (16.6%)	745 (79.7%)	*p* <0.001*∗*
Cigarette smoking			
No	564 (47.0%)	106 (11.3%)	
Yes	636 (53.0%)	829 (88.7%)	*p* <0.001*∗*
Alcohol drinking			
No	963 (80.3%)	440 (47.1%)	
Yes	237 (19.7%)	495 (52.9%)	*p* <0.001*∗*
Histological grade			
I+II		466 (49.8%)	
III+IV		469 (50.2%)	
Tumor status (T)			
T1+T2		540 (57.8%)	
T3+T4		395 (42.2%)	
Lymph node metastasis (N)			
N0		628 (67.2%)	
N1+N2+N3		307 (32.8%)	
Distal metastasis (M)			
M0		926 (99.0%)	
M1		9 (1.0%)	
Cell differentiation			
Well differentiated		139 (14.9%)	
Moderately or poorly differentiated		796 (85.1%)	

The Mann-Whitney U test or Fisher's exact test was used to compare values between healthy controls and patients with OSCC. *∗*  *p* value < 0.05 was considered statistically significant.

**Table 2:** Genotyping and allele frequency of *RETN* single nucleotide polymorphisms in oral squamous cell carcinoma and cancer-free, healthy males (controls).

Variable	Controls (N=1,200)	Patients (N=935)	OR (95% CI)	AOR (95% CI)
**rs3745367**				
GG	461 (38.4%)	349 (37.3%)	1.000 (reference)	1.000 (reference)
GA	561 (46.8%)	449 (48.0%)	1.057 (0.877-1.274)	1.132 (0.888-1.444)
AA	178 (14.8%)	137 (14.7%)	1.017 (0.782-1.322)	1.066 (0.755-1.506)
GA+AA	739 (61.6%)	586 (62.7%)	1.047 (0.878-1.249)	1.116 (0.887-1.406)
**rs7408174**				
TT	603 (50.3%)	499 (53.4%)	1.000 (reference)	1.000 (reference)
TC	497 (41.4%)	369 (39.5%)	0.897 (0.750-1.074)	0.891 (0.705-1.126)
CC	100 (8.3%)	67 (7.1%)	0.810 (0.581-1.128)	0.901 (0.588-1.380)
TC+CC	597 (49.7%)	436 (46.6%)	0.883 (0.744-1.047)	0.893 (0.714-1.116)
**rs1862513**				
GG	443 (36.9%)	352 (37.7%)	1.000 (reference)	1.000 (reference)
GC	575 (47.9%)	452 (48.3%)	0.989 (0.821-1.192)	0.991 (0.777-1.265)
CC	182 (15.2%)	131 (14.0%)	0.906 (0.696-1.181)	0.908 (0.644-1.280)
GC+CC	757 (63.1%)	583 (62.3%)	0.969 (0.812-1.157)	0.971 (0.771-1.223)
**rs3219175**				
GG	761 (63.4%)	590 (63.1%)	1.000 (reference)	1.000 (reference)
GA	397 (33.1%)	304 (32.5%)	0.988 (0.822-1.187)	0.918 (0.722-1.168)
AA	42 (3.5%)	41 (4.4%)	1.259 (0.808-1.962)	0.994 (0.559-1.767)
GA+AA	439 (36.6%)	345 (36.9%)	1.014 (0.849-1.210)	0.926 (0.735-1.168)

The adjusted odds ratios (AORs) with their 95% confidence intervals (CIs) were estimated by multiple logistic regression models that controlled for betel quid chewing, tobacco smoking, and alcohol consumption.

**Table 3:** Associations of the combined effect of *RETN* gene polymorphisms and betel nut chewing with the susceptibility to oral cancer among 1,465 smokers.

**Variable**	**Controls (n=636) (**%**)**	**Patients (n=829) (**%**)**	**OR (95**%** CI)**	**AOR (95**%** CI)**
**rs3745367**				
^a^GG genotype & non-betel nut chewing	178 (28.0%)	39 (4.7%)	1.00 (reference)	1.000 (reference)
^b^GA or AA genotype or betel nut chewing	135 (21.2%)	291 (35.1%)	**9.838 (6.580-14.708)**	**8.544 (5.668-12.880)**
^c^GA or AA genotype with betel nut chewing	323 (50.8%)	499 (60.2%)	**7.051 (4.852-10.246)**	**5.838 (3.985-8.552)**

**rs7408174**				
^a^TT genotype & non-betel nut chewing	218 (34.3%)	62 (7.5%)	1.00 (reference)	1.000 (reference)
^b^TC or CC genotype or betel nut chewing	127 (20.0%)	385 (46.4%)	**10.659 (7.540-15.068)**	**8.583 (6.026-12.225)**
^c^TC or CC genotype with betel nut chewing	291 (45.7%)	382 (46.1%)	**4.616 (3.349-6.361)**	**4.105 (2.960-5.692)**

**rs1862513**				
^a^GG genotype & non-betel nut chewing	166 (26.1%)	41 (5.0%)	1.00 (reference)	1.000 (reference)
^b^GC or CC genotype or betel nut chewing	138 (21.7%)	289 (34.9%)	**8.479 (5.698-12.617)**	**6.708 (4.467-10.074)**
^c^GC or CC genotype with betel nut chewing	332 (52.2%)	499 (60.1%)	**6.085 (4.208-8.800)**	**5.090 (3.493-7.418)**

**rs3219175**				
^a^GG genotype & non-betel nut chewing	287 (45.1%)	78 (9.4%)	1.00 (reference)	1.000 (reference)
^b^GA or AA genotype or betel nut chewing	125 (19.7%)	448 (54.0%)	**13.187 (9.583-18.147)**	**10.882 (7.861-15.064)**
^c^GA or AA genotype with betel nut chewing	224 (35.2%)	303 (36.6%)	**4.977 (3.672-6.746)**	**4.134 (3.028-5.644)**

The adjusted odds ratios (AORs) with their 95% confidence intervals (CIs) were estimated by multiple logistic regression models that controlled for alcohol consumption.

**Table 4:** Associations between the *RETN* rs3219175 polymorphism and clinical status in men aged >54 years with OSCC.

	**Clinical Stage**		OR (95% CI)	AOR (95% CI)^a^
*RETN* rs3219175	Stage I/II (n=243)	Stage III/IV (n=239)		
GG	160 (65.8%)	150 (62.8%)	1.000 (reference)	1.000 (reference)
GA	74 (30.5%)	73 (30.5%)	1.052 (0.711-1.558)	1.030 (0.693-1.529)
AA	9 (3.7%)	16 (6.7%)	1.896 (0.813-4.421)	1.896 (0.808-4.452)
	**Tumor size**			
*RETN* rs3219175	≤T2 (n=272)	> T2 (n=210)		
GG	181 (66.5%)	129 (61.4%)	1.000 (reference)	1.000 (reference)
GA	82 (30.2%)	65 (31.0%)	1.112 (0.748-1.653)	1.113 (0.747-1.658)
AA	9 (3.3%)	16 (7.6%)	**2.494 (1.069-5.820)** ^**b**^	**2.480 (1.058-5.814)** ^**c**^
	**Lymph node metastasis**			
*RETN* rs3219175	No (n=333)	Yes (n=149)		
GG	215 (22.8%)	95 (63.8%)	1.000 (reference)	1.000 (reference)
GA	102 (54.5%)	45 (30.2%)	0.998 (0.652-1.528)	0.969 (0.631-1.488)
AA	16 (22.7%)	9 (6.0%)	1.273 (0.543-2.983)	1.240 (0.525-2.930)
	**Metastasis**			
*RETN* rs3219175	M0 (n=477)	M1 (n=5)		
GG	308 (64.6%)	2 (40.0%)	1.000 (reference)	1.000 (reference)
GA	144 (30.2%)	3 (60.0%)	3.208 (0.530-19.411)	3.009 (0.491-18.451)
AA	25 (5.2%)	0 (27.2%)	-	0.783 (0.172-3.559)
	**Cell differentiation**			
*RETN* rs3219175	Well (n=76)	Moderate/poor (n=406)		
GG	51 (67.1%)	259 (63.8%)	1.000 (reference)	1.000 (reference)
GA	21 (27.6%)	126 (31.0%)	1.181 (0.681-2.050)	1.176 (0.675-2.047)
AA	4 (5.3%)	21 (5.2%)	1.034 (0.340-3.139)	0.963 (0.315-2.945)

^a^Multivariate analysis adjusted for the effects of betel quid chewing, tobacco smoking, and alcohol consumption.

^b^ *p *= 0.0345; ^c^*p *= 0.0367.

**Table 5:** *RETN* haplotype frequencies in healthy controls and OSCC patients.

Haplotype block				Controls	Patients		
**rs3745367 G/A**	rs7408174 T/C	rs1862513 G/C	rs3219175 G/A	n = 2400	n = 1870	OR (95% CI)	AOR (95% CI)^a^
G	T	G	G	679 (28.3%)	492 (26.3%)	1.000 (reference)	1.000 (reference)
G	C	G	G	475 (19.8%)	422 (22.6%)	1.226 (1.029-1.461)^b^	1.212 (0.965-1.522)
A	T	C	A	318 (13.2%)	291 (15.6%)	1.263 (1.037-1.538)^c^	1.150 (0.890-1.486)
A	T	G	G	257 (10.7%)	226 (12.1%)	1.214 (0.981-1.502)	**1.376 (1.039-1.823)** ^g^
A	T	C	G	189 (7.9%)	153 (8.2%)	1.117 (0.876-1.424)	1.219 (0.890-1.672)
G	T	C	G	191 (8.0%)	146 (7.8%)	1.055 (0.826-1.347)	1.062 (0.773-1.460)
G	T	C	A	67 (2.8%)	57 (3.0%)	1.174 (0.810-1.703)	1.051 (0.644-1.716)
A	C	C	A	86 (3.6%)	36 (1.9%)	0.578 (0.385-0.867)^d^	0.712 (0.427-1.188)
G	C	C	G	63 (2.6%)	30 (1.6%)	0.657 (0.419-1.031)	0.889 (0.509-1.552)
A	C	G	G	46 (1.9%)	14 (0.7%)	0.420 (0.228-0.773)^e^	0.343 (0.161-0.728)^h^
A	C	C	G	19 (0.8%)	1 (0.1%)	0.073 (0.010-0.544)^f^	0.176 (0.019-1.643)
G	C	C	A	6 (0.2%)	0 (0.0%)	-	-
A	T	G	A	2 (0.1%)	2 (0.1%)	1.380 (0.194-9.831)	1.620 (0.105-24.958)
G	C	G	A	2 (0.1%)	0 (0.0%)	-	-

^a^Multivariate analysis adjusted for the effects of betel quid chewing, tobacco smoking, and alcohol consumption.

^b^ *p*=0.0225; ^c^*p*=0.0201; ^d^*p*=0.0081; ^e^*p*=0.0053; ^f^*p*=0.0107; ^g^*p*=0.0258; ^h^*p*=0.0054.

OSCC: oral squamous cell carcinoma; OR: odds ratio; AOR: adjusted odds ratio.

**Table 6:** Combined effect of betel quid chewing and *RETN* haplotypes on OSCC development.

Betel quid chewing	*RETN *haplotype	Controls	Patients	AOR (95% CI)^b^
		n = 2400	n = 1870	
Yes	A-T-G-G	33 (1.4%)	179 (9.6%)	18.390 (12.333-27.421)^c^
Yes	Others^a^	365 (15.2%)	1311 (70.1%)	12.774 (10.666-15.300)^c^
No	A-T-G-G	224 (9.3%)	47 (2.5%)	1.203 (0.856-1.690)
No	Others^a^	1778 (74.1%)	333 (17.8%)	1.000 (reference)

^a^Other haplotypes included G-T-G-G, G-C-G-G, A-T-C-A, A-T-C-G, G-T-C-G, G-T-C-A, A-C-C-A, G-C-C-G, A-C-G-G, A-C-C-G, G-C-C-A, A-T-G-A, and G-C-G-A.

^b^Multivariate analysis adjusted for the effects of tobacco smoking and alcohol consumption.

^c^ *p*<0.001.

OSCC: oral squamous cell carcinoma.

## Data Availability

The data used to support the findings of this study are available from the corresponding author upon request.
